# Verordnungsverhalten von bayerischen Hausärzt*innen an der stationär-ambulanten Schnittstelle vor dem Hintergrund der Bayerischen Wirkstoffvereinbarung – Qualitative Ergebnisse der WirtMed-Studie

**DOI:** 10.1007/s00103-022-03563-6

**Published:** 2022-07-15

**Authors:** Nikoletta Zeschick, Julia Gollnick, Julia Muth, Franziska Hörbrand, Peter Killian, Wolfgang Krombholz, Norbert Donner-Banzhoff, Thomas Kühlein, Maria Sebastião

**Affiliations:** 1 Allgemeinmedizinisches Institut, Universitätsklinikum Erlangen, Friedrich-Alexander-Universität Erlangen-Nürnberg (FAU), Erlangen, Deutschland; 2 Abteilung für Allgemeinmedizin, Präventive und Rehabilitative Medizin, Philipps Universität Marburg, Marburg, Deutschland; 3 Kassenärztliche Vereinigung Bayerns, München, Deutschland

**Keywords:** Wirtschaftlichkeit, Hausärztliche Versorgung, Klinische Entscheidungsfindung, Intersektorale Zusammenarbeit, Wirtschaftlichkeitsgebot, Cost effectiveness, Primary healthcare, Clinical reasoning, Intersectoral collaboration, Efficiency principle

## Abstract

**Hintergrund und Ziel:**

Zur transparenten Steuerung der Arzneimittelausgaben im Rahmen des Wirtschaftlichkeitsgebots (§ 12 Fünftes Buch Sozialgesetzbuch (SGB V)) hat die Kassenärztliche Vereinigung Bayerns im Jahr 2014 die Wirkstoffvereinbarung (WSV) eingeführt. Diese hat die Richtgrößensystematik abgelöst. Mit Bezug auf die Rolle der WSV werden im Artikel die Gründe der Hausärzt*innen (HÄ) für oder gegen eine Weiterverordnung von Arzneimitteln aus dem Krankenhaus beschrieben.

**Material und Methode:**

In einem qualitativen Studiendesign wurden im Zeitraum 11/2019 bis 03/2020 mit bayerischen HÄ Einzelinterviews (*n* = 18) und 2 Fokusgruppen (*n* = 10) durchgeführt und nach der qualitativen Inhaltsanalyse ausgewertet.

**Ergebnisse:**

Mit der Einführung der WSV nahmen die Regresssorgen der HÄ insgesamt ab. Große Bedeutung bei Verordnungen haben – vor der Wirtschaftlichkeit – die patient*innenorientierte Versorgung und fachliche Richtigkeit von Therapieentscheidungen. Mit der Entlassmedikation ergeben sich wirtschaftliche Herausforderungen, besonders mit dem Leitsubstanzziel der oralen Antikoagulation, den Generikazielen bei Antidiabetika und bei Therapeutika für das Herz-Kreislauf-System. Allgemein kritisiert werden Rabattverträge, die oft zu Umstellungen von Arzneimitteln führen. Vereinzelt wird von einer „Vormachtstellung“ der Klinikärzt*innen berichtet, die dem hausärztlichen wirtschaftlichen Handeln entgegenstehen. Es fehlt laut HÄ eine sektorenübergreifende Kostenverantwortung.

**Diskussion:**

Ein reibungsloser Schnittstellenübergang ist aus Sicht der HÄ trotz des Rahmenvertrags Entlassmanagement und der neuen Steuerungssystematik der WSV im ambulanten Sektor noch nicht vorhanden. Für eine wirtschaftliche Arzneimittelversorgung bedarf es weiterhin einer sektorenübergreifenden, aber auch bundesländerübergreifenden politischen Diskussion.

**Zusatzmaterial online:**

Im Onlinematerial sind vertiefende Informationen zu der Methodik (Onlinematerial 1: Interview- und Fokusgruppenleitfaden) sowie zur Auswertung (Onlinematerial 2: Kategoriensystem) dieser Studie (10.1007/s00103-022-03563-6) verfügbar.

## Abstract (en)

**Background and objectives:**

In 2014, the Association of Statutory Health Insurance Physicians of Bavaria introduced the active substance agreement (WSV) for the transparent control of pharmaceutical expenditure within the framework of the efficiency principle (§ 12 of the Fifth Book of the German Social Code [SGB V]). It replaced the prescribing target scheme. Regarding the role of the WSV, the article presents the reasons of the general practitioners (GPs) for or against a further prescription of drugs from the hospital.

**Materials and methods:**

In a qualitative study design, individual interviews (*n* = 18) and two focus groups (*n* = 10) were conducted with Bavarian GPs between November 2019 and March 2020 and evaluated according to qualitative content analysis.

**Results:**

With the introduction of the WSV, recourse concerns decreased for GPs overall. Patient-oriented care and the professional correctness of therapy decisions are of great importance in prescribing, ahead of cost-effectiveness. Economic challenges arise with discharge medication, especially with the lead substance target of oral anticoagulants, the generic targets for antidiabetics, and for therapeutics for the cardiovascular system. Generally criticized are drug discount contracts, which often lead to drug changes after hospital discharge. There are individual reports of a “predominance” of hospital physicians when prescribing, which is contrary to their own economic actions as GPs. According to the GPs, there is a lack of cross-sectoral cost responsibility.

**Conclusions:**

In the view of GPs, a smooth interface transition is not yet available in the outpatient sector despite a framework agreement on discharge management and the new control system of the WSV. An economical supply of drugs continues to require political discussion across sectors, but also across federal states.

## Einleitung

In Deutschland haben gesetzlich Versicherte Anspruch auf eine Versorgung mit Arznei‑, Verband‑, Heil- und Hilfsmitteln gemäß §§ 31 ff. Fünftes Buch Sozialgesetzbuch (SGB V). Diese muss entsprechend dem Wirtschaftlichkeitsgebot ausreichend, zweckmäßig und wirtschaftlich sein (§ 12 SGB V). Zur Sicherstellung der ambulanten Versorgung treffen die Krankenkassen gemeinsam mit den kassenärztlichen Vereinigungen Arznei- und Heilmittelvereinbarungen (§ 84 und § 106b SGB V).

In Bayern gilt seit 2014 die *Wirkstoffvereinbarung* (WSV) zur Steuerung von Arzneimittelverordnungen, die die Richtgrößensystematik ablöste [1]. Die Kassenärztliche Vereinigung Bayerns (KVB) hat mit der WSV eine Steuerungssystematik implementiert, die das Ziel „steuern statt prüfen“ verfolgt, damit ein Regress nicht erfolgen muss [1]. Während die Richtgröße den für Arznei- sowie Heilmittelverordnungen pro Patient*in und Quartal im Durchschnitt zur Verfügung stehenden Eurobetrag widerspiegelte, basiert die WSV auf der Messgröße Defined Daily Doses (DDD, dt.: definierte Tagesdosen) und Zielwerten/Quoten in verschiedenen Wirkstoffgruppen [2, 3].

Die Steuerung der Arzneimittelverordnungen erfolgt hierbei unter Einbezug von [1, 4]: 1. Generika, welche als kostengünstige Alternative zu Originalpräparaten mit gleicher qualitativer und quantitativer Wirkstoffzusammensetzung gelten und nach Ablauf des Patentschutzes auf dem Markt verfügbar werden [5], 2. Leitsubstanzen, welche bei Arzneimitteln mit vergleichbarer Wirkung als wirtschaftlich festgelegt werden [6], und 3. Rabattverträgen zwischen pharmazeutischen Unternehmen und Krankenkassen mit zusätzlich finanziellem Einsparpotenzial [7]. Ärzt*innen sollen den Wirkstoff und die Menge in bestimmten Indikationsgruppen auswählen und Zielquoten erreichen (Tab. 1).

**Table 1:** 

Zielwerttabelle in % (Anteil Generika/Leitsubstanzen an Wirkstoffgruppe Gesamt)					
*Generikaziele ab 01.12.2016*^*a*^	Antidiabetika exkl. Insuline	Kombigruppe kardiovaskuläres System	Mittel bei obstruktiven Atemwegserkrankungen (Asthma, COPD)	Psycholeptika	Thrombozytenaggregationshemmer
Fachärzt*innen für Allgemeinmedizin, Allgemeinärzt*innen, praktische Ärzt*innen und hausärztliche Internist*innen	64,50	97,0	65,50	83,70	90,40
*Leitsubstanzziele ab 01.12.2016*^*a*^	Antikoagulanzien^b,c^	DOAKs^d^	TNF-Alpha-Blocker^b,e^	–	
Fachärzt*innen für Allgemeinmedizin, Allgemeinärzt*innen, praktische Ärzt*innen und hausärztliche Internist*innen	53,00	70,00	23,00		

*COPD* Chronisch obstruktive Lungenerkrankung, *DOAK* direkte orale Antikoagulanzien, *TNF* Tumornekrosefaktor

^a^ Insgesamt 24 Generikaziele und 6 Leitsubstanzziele

^b^ Rabattierte Nichtleitsubstanzen zählen als unwirtschaftlich i. S. dieser Vereinbarung

^c^ Leitsubstanzen = Warfarin, Phenprocoumon

^d^ Leitsubstanzen = Apixaban, Edoxaban, Rabattarzneimittel

^e^ Leitsubstanzen = Benepali®, Flixabi®, Inflectra®, Remsima®

**Figure 1:**
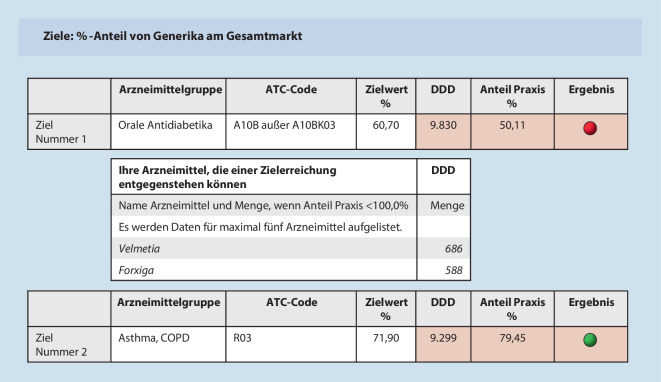

Niedergelassene Ärzt*innen erhalten von der KVB quartalsweise eine Arzneimitteltrendmeldung (AMTM) als Frühinformation. In der AMTM erhalten sie Rückmeldung zu ihrem Verordnungsverhalten und die Erreichung der Generika- und Leitsubstanzzielquoten. Die Darstellung in der AMTM erfolgt anhand einer Ampel. Zusätzlich wird den Ärzt*innen Feedback aufgezeigt, welche Arzneimittel einer Zielerreichung entgegenstehen können (Abb. 1). Über alle verordneten Indikationsgruppen wird ein kumuliertes und ein gewichtetes Gesamtergebnis berechnet. Bei Mindest- bzw. Übererfüllung der Zielquoten errechnet sich eine Gesamtzielerreichung von ≥ 100 %, welche in jedem Fall von einer Arzneimittelprüfung für das entsprechende Quartal befreit.

Bezüglich der Kostensteuerung liegt der Fokus auch immer wieder auf dem Sektorenübergang stationär-ambulant. Wie Studien der letzten 20 Jahre zeigen, wirken sich Krankenhausverordnungen auf das ambulante Verordnungsverhalten aus [9–13]. So wird beispielsweise bei Krankenhausaufnahme die bestehende Medikation häufig umgestellt [9]. Nach Entlassung kommt es bei bis zu 84 % der Patient*innen erneut zu Medikationsveränderungen [11]. Medikamentenbudgets spielen hierbei, laut einer Interviewstudie aus dem Jahr 2013, bei 80 % der niedergelassenen Hausärzt*innen (HÄ) eine Rolle [12]. Dies liegt unter anderem daran, dass eine wirtschaftliche Versorgung für das Krankenhaus nicht zwangsläufig auch eine wirtschaftliche Versorgung im ambulanten Sektor bedeuten muss, z. B. aufgrund unterschiedlicher Bezugspreise der Apotheken [14].

Das Projekt *WirtMed – die Verordnung von Arzneimitteln: Prüfung und Steuerung von Wirtschaftlichkeit und Qualität* ist gefördert vom Innovationsfonds des Gemeinsamen Bundesausschusses (G-BA; [15]). Es hat zum Ziel, die unterschiedlichen Prüf- und Steuerverfahren von Arzneimittelverordnungen zu beleuchten und die Ergebnisse zu nutzen, um die bereits bestehenden Regularien zum Zweck der Kostenkontrolle weiterzuentwickeln. Das hier beschriebene Teilprojekt der WirtMed-Studie untersucht, wie die niedergelassene Ärzteschaft die WSV bewertet, welche Erfahrungen mit ihr bestehen und zu welchem Verordnungsverhalten sie führt [16]. Dies beinhaltet auch die Reaktion auf Verordnungen von ärztlichen Kolleg*innen. Der vorliegende Artikel fokussiert auf den Umgang der bayerischen HÄ mit der Entlassmedikation vor dem Hintergrund der Einführung der WSV.

## Methoden

### Studiendesign

Im Zeitraum 11/2019 bis 03/2020 wurden qualitative, teilstrukturierte Einzelinterviews mit 18 HÄ und 2 Fokusgruppen mit 10 HÄ durchgeführt.

Die Interviews zielten darauf ab, Details zum ärztlichen Umgang mit der WSV zu erfassen. Anschließend erfolgten Fokusgruppen, in denen genannte Beweggründe diskutiert und weitere Ansichten und Meinungen diesbezüglich gesammelt wurden.

### Rekrutierung und Setting

Es erhielten 697 HÄ in Bayern mit ihrer AMTM ein schriftliches Einladungsschreiben zur Studienteilnahme. HÄ waren Fachärzt*innen für Allgemeinmedizin, hausärztlich tätige Internist*innen und praktische Ärzt*innen. Einschlusskriterium für die Interviews war der Parameter „Erreichung aller Wirkstoffziele von ≥ 90 % in den Quartalen des Jahres 2017“. Der nachfolgende Kontakt erfolgte ausschließlich über das Allgemeinmedizinische Institut des Universitätsklinikums Erlangen. Die Interviews führten jeweils 2 Mitarbeiterinnen in der jeweiligen Arztpraxis, ebenso die Fokusgruppen, welche im Institut oder in einem angemieteten Raum stattfanden. Es wurden alle HÄ befragt, die ihr Interesse an der Studienteilnahme erklärten.

### Datenerhebung

Der Interviewleitfaden wurde an den universitären Einrichtungen Erlangen und Marburg entwickelt (Pädagogin, Psychologin sowie Apothekerin) und mit 4 Ärzt*innen vorab getestet. Er umfasste 4 Themenblöcke: allgemeine Informationen zu Person/Praxiserfahrung (z. B. Dauer ambulanter Tätigkeit), Allgemeines zum Verordnungsverhalten (z. B. Umgang mit von ärztlichen Kolleg*innen initiierten Medikamenten, auch explizit Entlassmedikation), Gesamtsituation bezüglich WSV sowie Abschluss und Fazit (s. Onlinematerial 1). Interviewerinnen und HÄ kannten sich zuvor nicht. Nach schriftlicher Einverständniserklärung wurden die Interviews (ca. 60 min) und Fokusgruppen (ca. 120 min) als Digitalaudio aufgezeichnet und schriftliche Protokolle erstellt.

### Datenauswertung

Die Audiodateien wurden vollständig nach Dresing und Pehl [17] transkribiert und anonymisiert. Die Auswertung erfolgte anhand der qualitativen Inhaltsanalyse nach Mayring (durch die Erstautorin NZ), mittels eines deduktiv-induktiven Vorgehens und mithilfe des Softwareprogramms MAXQDA Plus 2020 (VERBI-GmbH, Berlin, Deutschland) [18]. Initial wurde anhand des Interviewleitfadens ein Kategoriensystem gebildet. Dieses wurde induktiv um Kategorien und -ausprägungen erweitert (s. Onlinematerial 2). Für die konsensuelle Validierung der Kategorien wurden diese fortlaufend im Projektteam diskutiert. Nach der Hälfte der Interviewauswertung kamen keine neuen Kategorien hinzu. Alle Transkripte wurden anschließend anhand des finalen Kategoriensystems codiert. Die Ergebnisdarstellung folgt den Kategorien und Zitate werden anhand vergebener Kürzel und Absatzangabe belegt (z. B. TN-03:83).

Eine kommunikative Ergebnisvalidierung erfolgte am Erlanger *Tag der Allgemeinmedizin 2020*. Dabei wurden erste Analyseergebnisse visualisiert und mit bayerischen HÄ abgesichert. Die Berichterstattung erfolgt nach der COREQ-Checkliste (*Consolidated Criteria for Reporting Qualitative Research;* [19]).

## Ergebnisse

An der Studie nahmen 28 HÄ (19 Männer, 9 Frauen) diverser Altersgruppen teil (Tab. 2). Vertreten waren sowohl Einzel- und Gemeinschaftspraxen als auch verschiedene Praxisstandorte (Land, Klein‑, Mittel- und Großstadt).

**Table 2:** 

	Gesamt (*n* = 28)		Männer (*n* = 19)		Frauen (*n* = 9)	
	*n*	%	*n*	%	*n*	%
*Alter*						
41–50 Jahre	11	37	7	37	4	45
51–60 Jahre	7	26	5	26	2	22
Ü. 60 Jahre	10	37	7	37	3	33
*Praxisart*						
Einzelpraxis	14	50	7	37	7	78
Gemeinschaftspraxis	11	39	9	47	2	20
Sonstiges^a^	3	11	3	16	1	10
*Praxislage*						
Land^b^	8	29	5	26	3	33
Klein‑/Mittelstadt^c^	11	39	9	47	2	22
Großstadt^d^	9	32	5	26	4	45
*Beschäftigungsart*						
Vollzeit	25	89	17	90	8	89
Teilzeit	3	11	2	10	1	11

^a^Praxisgemeinschaft, Einzelpraxis mit angestellten Ärzt*innen

^b^Einwohnerzahl unter 5000

^c^Einwohnerzahl unter 100.000

^d^Einwohnerzahl über 100.000

Die HÄ kommen insgesamt mit der WSV gut zurecht. Die Schnittstelle stationär-ambulant stellt für die HÄ eine Herausforderung dar, sowohl bezüglich der Kontinuität der Patient*innenversorgung mit Arzneimitteln als auch der Wahrung der Wirtschaftlichkeit bei der Verordnung dieser. Die HÄ thematisieren den Umgang mit Entlassmedikation oft von sich aus und mit emotionaler Färbung. Beides hebt die Relevanz des Themas hervor.

Umstellungen, aber auch das Beibehalten der Entlassmedikation, betreffen sowohl Neueinstellungen als auch in der Klinik vorgenommene Umstellungen (TN-03:83; TN-05:121; TN-15:26; TN-01:32). Insgesamt konzentrieren sich die HÄ in ihren Ausführungen mehr auf Neuverordnungen als auf im Krankenhaus vorgenommene Umstellungen. Nur eine HÄ geht nicht auf Neuverordnungen ein, sondern berichtet nur, dass ihre Patient*innen in der Klinik nicht umgestellt werden.

### Gründe für und gegen eine Weiterverordnung von Arzneimitteln

Es zeigt sich ein heterogenes Bild mit vielschichtigen Gründen, warum Medikamente beibehalten oder umgestellt werden. Übergeordnet sind wichtig: 1. die patient*innenorientierte Versorgung, 2. die Berücksichtigung des Arbeitsaufwandes, 3. die eigenen Annahmen und 4. die Wirtschaftlichkeit. Die HÄ gewichten diese einzelnen Faktoren unterschiedlich stark, wobei viele den Fokus auf die patient*innenorientierte Versorgung legen.

#### Patient*innenorientierte Versorgung

Bei der patient*innenorientierten Versorgung stehen die Adhärenz und somit das (psychische wie physische) Patient*innenwohl im Fokus. HÄ betonen die langjährige Patient*innenbegleitung und deren Auswirkung auf die Medikamentenverordnung (TN-15:32). Je nach Patient*in können kostengünstige, dafür den Patient*innen altbekannte oder teure, neue Medikamente gewünscht sein (TN-20:19; TN-01:31): „Dann sind die auch psychisch drauf fixiert, dass die auch wirklich tatsächlich die Originalpräparate haben wollen. … Und das kann man dann auch nicht umstellen“ (TN-08:60). Die Patient*innenerwartung an die HÄ spielt eine wichtige Rolle im Verordnungsverhalten: Manche nehmen dafür sogar rote Punkte in der AMTM (Abb. 1), im Sinne einer individuellen Unwirtschaftlichkeit, in Kauf (TN-17:127).

#### Berücksichtigung des Arbeitsaufwandes

Das Weiterverordnen der Entlassmedikation wird allerdings auch als Wertschätzung der Klinikarbeit gesehen (TN-15:26). Hier wurde Zeit investiert, die bei einer Umstellung umsonst gewesen wäre. Gleichzeitig sehen die HÄ auch den eigenen Zeitaufwand der Medikationseinstellung bzw. -umstellungen (TN-02:442). Vor allem bezüglich der direkten oralen Antikoagulanzien (DOAKs) ist eine (Rück‑)Umstellung auf Phenprocoumon „nicht umsetzbar“ (TN-20:51), da dies „eine riesen Diskussionsarbeit“ ist (TN-06:69). Hier zeigt sich eine gewisse Dominanz der Klinik, die eine Behandlung vorgibt, die ambulant schwer zu ändern ist. Die HÄ stehen im Spannungsverhältnis zwischen den Vorschlägen der Klinik und der eigenen Wirtschaftlichkeit: „Wir versuchen jetzt wenigstens dahin zu gehen, dass wir es steuern und mehr Eliquis und Lixiana verschreiben, … weil die [Kliniken] immer Xarelto aufschreiben“ (TN-03:75). In diesem Spannungsverhältnis fühlen sich manche HÄ hilflos, was zu einem Beibehalten der Entlassmedikation und damit auch zu roten Punkten in der AMTM (Abb. 1) führen kann.

#### Eigene Annahmen

Zudem kann die persönliche Präferenz – Was würde ich selbst einnehmen? – als Behandlungsmaßstab dienen (TN-10:99) und zur Weiterverordnung unwirtschaftlicher Medikamente führen. HÄ berichten auch, dass sie der Fachkompetenz und Integrität der Klinikärzt*innen insgesamt vertrauen; entsprechende Verordnungen werden dann weitergeführt (TN-05:123).

Viele HÄ sind allerdings auch kritisch gegenüber der Pharmaindustrie und folglich bei neuen, teuren Medikamenten (TN-20:43; TN-3:259). Sie verordnen entsprechende Medikamente nicht weiter. Ebenfalls berichten sie, dass DOAKs stark beworben und im Extremfall die Verordnung von Vitamin-K-Antagonisten (VKAs) sogar von Meinungsbildnern, wie im Krankenhaus tätigen Kardiolog*innen, als „Kunstfehler“ dargestellt werden (TN-11:65; TN-13:47).

Wenn HÄ die Nachvollziehbarkeit oder Begründung fehlt, stellen sie Verordnungen um: „Also oft macht [das Krankenhaus] einen anderen Blutdrucksenker rein … völlig grundlos“ (TN-05:123).

#### Wirtschaftlichkeit

Ein Kostenbewusstsein ist den meisten HÄ selbstverständlich, um das Solidarsystem zu wahren (TN-03:283). Unter Wirtschaftlichkeit verstehen viele der Ärzt*innen insbesondere den Einsatz von Generika. Gleichzeitig sprechen sie oftmals von einer „vernünftigen“ (TN-09:168) Therapie, die sich an Patient*innen orientiert: „Ich glaube, diese ganzen wirtschaftlichen Fragen sind schon schön und gut. Aber am Ende arbeitet man mit dem Patienten“ (TN-07:184). Nur wenige berichten davon, Wirtschaftlichkeit in ihrer Verordnungsentscheidung überzuordnen: „Letztendlich ordnet sich alles unter die Wirtschaftlichkeit“ (TN-07:79).

Im Vergleich zum stationären Sektor sehen die HÄ die eigenen Arzneimittelverordnungen als kleineren Kostenposten (TN-23:267). Teure Medikamente werden im Krankenhaus erstverordnet (TN-15:26). Der ambulante Sektor muss anschließend die Umstellung auf kostengünstigere Präparate vornehmen (TN-15:26)*. *Für einzelne HÄ ist es ein zentrales Anliegen, dass die Krankenhäuser als „primäre Weichensteller“ (TN-20:51) stärker in die Pflicht genommen werden sollen.

### Rolle der Wirtschaftlichkeitsvorgaben unter der WSV

#### WSV-Zielquoten von Generika, Leitsubstanzen und Rabattarzneimitteln

Die Quoten von Generika und Leitsubstanzen im Rahmen der WSV bereiten den HÄ grundsätzlich keine Schwierigkeiten und eine Trennung wird in den Interviews meist nicht vorgenommen. HÄ kategorisieren allerdings in ihren Aussagen die Arzneimittel oft in günstig und teuer, wobei sie günstig mit Generika verbinden: „Es kommt gelegentlich mal vor, dass ich ein Medikament für zu teuer halte, weil es ein ähnliches [günstigeres Blutdruckmedikament] gibt“ (TN-15:32). Im Krankenhaus verordnete Generika werden auch ambulant weiterverordnet: „Die kriegen normalerweise schon das, was aus dem Krankenhaus kommt. Außer es ist halt nicht generisch. Aber ansonsten übernehme ich das schon“ (TN-07:27).

Zudem berichten HÄ, bei ihren Verordnungen Patient*innen auf Rabattverträge umzustellen (TN-08:206). Gleichzeitig kritisieren sie aber die Intransparenz der Rabattverträge unabhängig von der Entlassmedikation. Es zeichne sich ein Gefühl des Ausgeliefertseins gegenüber den Krankenkassen ab: „Sie [die Krankenkassen] machen einen Rabattvertrag. ... Und wenn wir nicht ankreuzen, sind wir böse. Die bösen Ärzte, die nicht machen wollen, was die [anderen] Ärzte, beziehungsweise die Krankenhäuser sagen“ (TN-22:211). Manche HÄ stellen auch das Kosten-Nutzen-Verhältnis der Verträge infrage (TN-06:19). Eine mögliche (Rück‑)Umstellung der Entlassmedikation betrifft neben den kritisierten Rabattarzneimitteln dennoch auch Generika und Leitsubstanzen.

HÄ berichten auch von Problemen mit spezifischen Arzneimittelgruppen. Am häufigsten sprechen sie das Leitsubstanzziel der Arzneimittelgruppe der DOAKs an. Sie schildern ein deutliches Überwiegen der Verordnungen von DOAKs gegenüber Phenprocoumon bei Krankenhausentlassungen (TN-10:99), vor allem bei Erstverordnungen, aber auch bei Umstellungen. Nur wenige geben an, aktiv gegenzusteuern, indem sie auf Phenprocoumon umstellen. Mehrere berichten, auf die in der WSV vereinbarten Leitsubstanzen Apixaban und Edoxaban oder Rabattverträge umzustellen (TN-08:206; TN-03:75). Dennoch erreicht keiner der befragten HÄ den Zielwert bei den Antikoagulanzien.

Des Weiteren werden Schwierigkeiten beim Erreichen der Generikaziele in folgenden Arzneimittelgruppen genannt: Antidiabetika (TN-011:8), Therapeutika für das Herz-Kreislauf-System sowie die Indikationsgruppen COPD-/Asthmamittel und Psychopharmaka. Beispielsweise werden Medikamentenumstellungen rückgängig gemacht, wenn „der andere [Blutdrucksenker] zehn Jahre funktioniert hat“, wohingegen kardiologische Neueinstellungen nach genauer Abwägung auch beibehalten werden (TN-05:121).

Ebenso von den HÄ erwähnt werden Arzneimittel in Leitsubstanzzielen, wie z. B. das Ziel für die TNFα-Inhibitoren (Tumornekrosefaktor-Alpha-Blocker) zur Behandlung der rheumatoiden Arthritis. Deren Verordnung führt ebenfalls teilweise zu nicht erreichten Zielquoten und die Medikation wird entsprechend häufig nach dem Krankenhausaufenthalt umgestellt. Bei der Therapie mit COPD-/Asthmamitteln und Psychopharmaka hingegen ist die Umstellung für HÄ deutlich erschwert, da z. B. der Patient*innenwunsch nach Originalpräparaten (TN-08:60) oder die Handhabbarkeit bei Inhalativa im Vordergrund steht: „Wenn der ein Alvesco [zur Asthmabehandlung] hat, worauf soll ich denn den umsetzen? Jetzt atmet der gut, dann setze ich den doch nicht um, damit er einen Rabattvertrag hat“ (TN-03:103)*.*

#### Verschieben von Verschreibungen

Überweisungen zur finanziellen Entlastung spielen eine untergeordnete Rolle. Sehr selten berichten die HÄ, dass sie die Verordnungsmenge mit anderen Fachärzt*innen teilen (TN-13:47) oder für die größere Packungsgröße an diese verweisen (TN-08:60). Den HÄ ist bewusst, dass die Behandlungskosten auch bei Überweisung der Patient*innen gesamtwirtschaftlich gleichbleibend sind. Manche Patient*innen gehen auch selbst zu Fachärzt*innen, weil sie dort die teureren Medikamente verschrieben bekommen (TN-22:211).

#### Regresssorgen

Mehrheitlich wird in den Interviews berichtet, dass mit der Einführung der WSV die Regresssorgen abnahmen. Vor dem Hintergrund der WSV lassen sich 3 verschiedene Typen von Ärzt*innen ableiten: HÄ mit geringen Regresssorgen, mit größeren Regresssorgen sowie die Resignierten. Regresssorgen betreffen übergreifend alle Verordnungen, dennoch zeigt sich im Speziellen je nach Typ ein unterschiedlicher Umgang mit der Entlassmedikation.

Der Großteil der HÄ hat geringe Regresssorgen. Diese Gruppe vertraut in die WSV; auch vereinzeltes Nichterreichen von Zielwerten durch Entlassmedikamente bereitet ihnen keine Sorgen (TN-10:81). Für den Fall eines Prüfverfahrens wird die Weiterverordnung durch eine gute Dokumentation und den Krankenhausbericht abgesichert (TN-10:79).

HÄ mit größeren Regresssorgen begründen ihre (Rück‑)Umstellungen der Entlassmedikation damit, dass es zu vielen teuren Medikamenten gleichwertige Alternativen gibt. Größere Regresssorgen gehen mit einer ausführlicheren Prüfung der Entlassmedikation und ihrer Alternativen einher (TN-07:79). Umstellungen betreffen beispielsweise die Bluthochdrucktherapie, inklusive teurerer Kombinationspräparate: „Das [Kombinationspräparat] war genau das, was er vorher hatte. Nur [die Einzelsubstanzen, die] er vorher hatte, haben ein Drittel so viel gekostet [und kannte der Patient bereits jahrelang]“ (TN-05:123). Die Entlassmedikamente nicht weiter zu verordnen führt laut HÄ in den meisten Fällen zum Erreichen des Zielwertes.

Eine chronische Hilflosigkeit zeigt sich bei der dritten, kleinsten Gruppe: „Da war ich eine Zeit lang grün, jetzt bin ich langsam gelb. … Letztendlich, bei der Prüfung, muss man sagen, das ist nicht umsetzbar“ (TN-20:51). Dementsprechend resignieren manche und verordnen teure Medikamente aus dem Krankenhaus weiter. „Und ich sage Ihnen ganz ehrlich. Also inzwischen interessiert mich so ein [roter] Punkt … schon fast relativ wenig“ (TN-19:30).

## Diskussion

Die von HÄ genannten Gründe für oder gegen eine Weiterverordnung von Arzneimitteln aus dem Krankenhaus reichten von patient*innenorientierter Versorgung und dem benötigten Zeitaufwand über Vertrauen in die Krankenhausärzt*innen bis zu einem unterschiedlich ausgeprägten Fokus auf Wirtschaftlichkeitsaspekte.

### Patient*innenorientierte Versorgung, Wertschätzung der Arbeit

Patient*innenorientierte Versorgung nimmt einen hohen Stellenwert bei Medikamentenverordnungen ein und kann auch dazu führen, dass weniger Medikamente verschrieben werden [20, 21]. Das Spannungsverhältnis zwischen Patient*innenwohl und Wirtschaftlichkeit wurde bisher insbesondere im Kontext der Krankenhäuser untersucht, seltener in der ambulanten Versorgung [22, 23]. Interessant ist, dass den HÄ zum Teil nicht bekannt war, warum bestimmte Medikamente im Krankenhaus umgestellt wurden. Ein besserer Austausch scheint daher immer noch unabdingbar. Für eine reibungslose Schnittstellenzusammenarbeit sind beide Seiten bedeutsam. Medikationspläne sind für Haus- und Krankenhausärzt*innen gleichermaßen wichtig, um Medikationsumstellungen zu vermeiden.

Eine Verbesserung der Kommunikation (Übermittlung von Daten), Kooperation (Möglichkeit von Nachfragen/Rückmeldungen) und Koproduktion (gemeinsame Planung/Zielsetzung) sind dringend nötig. Dies sollte unter anderem EDV-unterstützt erreicht werden [24–26]. Es gibt zahlreiche Vorschläge zu sektorenübergreifenden Lösungen, wie z. B. Prozess- und Strukturvorgaben und eine Erweiterung der Handlungsmöglichkeiten. Neben dem Anspruch auf einen Medikationsplan gemäß § 31a Abs. 1 S. 1 SGB V i. V. m. § 29a Bundesmantelvertrag – Ärzte (BMV-Ä) sowie der Etablierung des elektronischen Medikationsplans könnten konsequente Netzwerktreffen und gemeinsame Behandlungspfade zielführend sein [27–29]. Bisherige Lösungsvorschläge waren nicht immer erfolgreich [27, 30]. Nur selten wird in der Literatur von der Wertschätzung klinischer Arbeit berichtet [31, 32]. Vielmehr spielt die Zeitnot eine Rolle, sodass „die Perspektive und Situation des Senders [gerade nicht] wertschätzend“ einbezogen wird [32]. Ärzt*innen beider Sektoren kritisieren sich teils gegenseitig [31].

Niedergelassene Ärzt*innen haben nur einen begrenzten Handlungsspielraum. Eine systempolitische Lösung wäre es, die Krankenhäuser gleichermaßen in die Pflicht zu nehmen. Die gesetzlichen Grundlagen hierfür gibt es: § 39 Abs. 1a SGB V und § 4 Abs. 2 Rahmenvertrag Entlassmanagement. Der Rahmenvertrag sieht einen Entlassbrief vor. Dabei soll dieser unter Berücksichtigung von § 115c SGB V Therapievorschläge unter Verwendung der Wirkstoffbezeichnung enthalten und die begonnene Arzneimitteltherapie auch in der vertragsärztlichen Versorgung zweckmäßig und wirtschaftlich sein. Um einen Standard für den Entlassbrief zu schaffen, wurde am 01.01.2022 der digitale Krankenhaus-Entlassbrief innerhalb der elektronischen Patientenakte von der Kassenärztlichen Bundesvereinigung und der Deutschen Krankenhausgesellschaft eingeführt [33]. Eine anhaltende Weiterentwicklung ist angedacht [33].

### Wirtschaftlichkeitsvorgaben und Regresssorgen

Prinzipiell sehen die HÄ Wirtschaftlichkeit als wichtig für das Solidarsystem an. Mit Ausnahme des Leitsubstanzziels „Antikoagulanzien“ (DOAKs) gaben die befragten HÄ an, dass sie nach Krankenhausentlassung häufig Medikamente umstellen oder neuverordnen. Dies betraf sowohl Generika als auch Leitsubstanzen.

Die HÄ berücksichtigen Rabattverträge, kritisieren sie aber zugleich auch. Rabattverträge nehmen eine bedeutende Rolle in der Steuerung der Arzneimittelausgaben ein [34]. Es muss jedoch beachtet werden, dass neue Verträge zu Präparatumstellungen führen, was den Gesprächs- und Aufklärungsbedarf der Patient*innen bezüglich der korrekten Medikamenteneinnahme erhöht [35]. Nach einem Krankenhausaufenthalt obliegt es zudem den HÄ und nicht den Klinikärzt*innen, die Patient*innen auf ein Rabattarzneimittel einzustellen. Eine zentrale Rolle bei der Aushändigung des (rabattierten) Medikamentes hat insbesondere die Apotheke [36, 37].

Quoten von Generika oder Leitsubstanzen bereiten den HÄ keine grundsätzlichen Probleme. Schwierigkeiten in der Einhaltung der Vorgaben bestanden insbesondere bei dem Leitsubstanzziel der DOAKs, gefolgt von den Generikazielen bei Antidiabetika und bei Therapeutika für das Herz-Kreislauf-System. Bei Letzterem sind die Zielwerte für die Kombinationspräparate oftmals schwer einzuhalten. Dies kann daraus resultieren, dass in Leitlinien beispielsweise Kombinationspräparate empfohlen werden, die nicht immer standardmäßig in der WSV vorgesehen sind. Die Verschreibung solcher ist in der *Kombigruppe kardiovaskuläres System* möglich, dabei besteht für die HÄ eine Generikazielquote von 97 % [8]. Möglicherweise ist es bezüglich der Verordnungsrichtlinien sinnvoll, bei Patient*innen mit einer hohen Anzahl an Medikamenten auch darüber hinaus Kombinationspräparate standardmäßig zu ermöglichen und Sonderregeln einzuführen.

Medikamentenumstellungen können zum Vertrauensverlust von Patient*innen gegenüber HÄ führen; mutmaßlich wirtschaftliche Umstellungen stehen besonders im Verdacht [38]. Diese Problematik betrifft vermutlich nicht nur HÄ, sondern den gesamten ambulanten Sektor. Dies spricht für die Einführung sektorenübergreifender Regelungen, die beispielsweise ein gemeinsames Budget für Patient*innen mit oraler Antikoagulation vorsehen.

Im Falle der DOAKs zeigen bisherige Studien keine eindeutige oder wesentliche Überlegenheit dieser gegenüber VKAs [39–42]. Erst seit Ende 2019 führt der Leitfaden der Arzneimittelkommission der deutschen Ärzteschaft (AkdÄ) aus, dass die Verordnung eines DOAKs auch „vertretbar“ ist, nachdem, bis auf Ausnahme von Edoxaban, Antidota (Gegenmittel) zugelassen sind [43]. Die klinische Wirksamkeit dieser kann jedoch noch nicht sicher beurteilt werden [43]. Unter Berücksichtigung der Blutgerinnungskontrolle mittels INR-Wert ist der Kostenfaktor ein Vorteil der VKAs [44]. Wenige HÄ nehmen den hohen Zeitaufwand für die Umstellung noch in Kauf, wobei es ihnen vorrangig nicht um die Richtigkeit der Medikation geht, sondern um die Wirtschaftlichkeit. Gleichzeitig besteht das Zeitproblem auch bei Krankenhausärzt*innen.

Die WSV hat die Regresssorgen vermindert. Auch in anderen Bundesländern wurde die Richtgrößenprüfung durch verschiedene Systematiken ersetzt [45]. Beispielsweise gilt in Hamburg, ähnlich wie in Bayern, eine WSV [46], in Baden-Württemberg gilt die Richtwertsystematik [47]. Während das hohe Alter, die Anzahl an Patient*innen und an Vorerkrankungen im alten Prüfsystem insbesondere HÄ in die Prüfzone bzw. den Regress führten [48], kommt eine Wirtschaftlichkeitsprüfung anhand der Wirkstoffauswahl, -menge und dem Verordnungsanteil, wie sie im Rahmen der WSV geregelt ist, den HÄ zugute. Anfang 2020 wurde die WSV überarbeitet, um den sich verändernden Versorgungsrealitäten zu folgen [49]. In dieser wurden beispielsweise die Zielquoten für DOAKs aufgrund der veränderten Marktsituation und Versorgungsrealität abgesenkt. Es ist noch offen, welche Auswirkungen dies auf die Verordnungen der Entlassmedikation hat.

### Limitationen

HÄ, die über mehrere Quartale hinweg eine Gesamtzielerreichung ≤90 % hatten, blieben unberücksichtigt. Diese wurden in einem anderen WirtMed-Teilprojekt befragt und machen lediglich rund 1 % aller bayerischen HÄ aus. Die KVB-Trendmeldungen wurden von den HÄ vorgelegt und/oder mündlich mitgeteilt. Sie wurden nicht inhaltlich analysiert. Die qualitative Studie spiegelt die subjektiven Wahrnehmungen der befragten HÄ wider. Wie viele Medikamente bei Krankenhausentlassung tatsächlich neu eingestellt oder umgestellt wurden (z. B. aufgrund des hohen Preises), kann nicht beantwortet werden. Den HÄ wurde in den Interviews der Raum gegeben, ihre Meinung und Einstellung zur intersektoralen Zusammenarbeit und der Entlassmedikation zu äußern. Sie legten hiermit ihren eigenen Fokus und bestärkten den explorativen Charakter der qualitativen Befragung.

## Fazit

Die HÄ stehen auch unter der WSV noch im Spannungsverhältnis zwischen ökonomischen Vorgaben, Patient*innenwunsch und guter kollegialer Zusammenarbeit. Herausforderungen ergeben sich besonders beim Übergang vom stationären zum ambulanten Sektor in der medizinischen Versorgung aufgrund der jeweiligen, zum Teil sehr unterschiedlichen Regelungen. Wirtschaftlich herausfordernd für die HÄ erscheinen insbesondere die Verordnungen von DOAKs sowie Antidiabetika. Medikamentenumstellungen sind nicht zuletzt auch aufgrund mangelnder intersektoraler Kommunikation häufig und können zum Vertrauensverlust bei Patient*innen führen, da hier mutmaßliche wirtschaftliche Umstellungen besonders im Verdacht stehen. Diese Problematik betrifft vermutlich nicht nur HÄ, sondern den gesamten ambulanten Sektor und spricht für die Einführung sektorenübergreifender Regelungen.

## Supplementary Information




