# Supplementary material for: Prescribing behavior of Bavarian general practitioners at the inpatient–outpatient interface within the context of the Bavarian active substance agreement—qualitative results of the WirtMed Study
Source: Bundesgesundheitsblatt Gesundheitsforschung Gesundheitsschutz. 2022 Jul 15;65(9):900–8. [Article in German] doi: 10.1007/s00103-022-03563-6 (PMC9436878; doi:10.1007/s00103-022-03563-6)
Supplement: Supplementary file 1 [file 103_2022_3563_MOESM1_ESM.pdf]

Bundesgesundheitsblatt – Gesundheitsforschung – Gesundheitsschutz, Ausgabe 9/2022

Onlinematerial zum Beitrag:

Verordnungsverhalten von bayerischen Hausärzt*innen an der stationär-ambulanten Schnittstelle vor dem Hintergrund der Bayerischen Wirkstoffvereinbarung – Qualitative Ergebnisse der WirtMed-Studie

Nikoletta Zeschick[1], Julia Gollnick[2], Julia Muth[2], Franziska Hörbrand[3], Peter Killian[3], Wolfgang Krombholz[3], Norbert Donner-Banzhoff[2], Thomas Kühlein[1], Maria Sebastião[1]

[1] Friedrich-Alexander-Universität Erlangen-Nürnberg (FAU), Allgemeinmedizinisches Institut, Erlangen, Deutschland
[2] Philipps Universität Marburg, Abteilung für Allgemeinmedizin, Präventive und Rehabilitative Medizin, Marburg, Deutschland
[3] Kassenärztliche Vereinigung Bayerns, München, Deutschland

Korrespondenzadresse

Dr. PH Maria Sebastião
Forschungskoordination
Universitätsklinikum Erlangen
Allgemeinmedizinisches Institut
Universitätsstr. 29
91054 Erlangen
Deutschland
Maria.Sebastiao@uk-erlangen.de

Inhalt:

Onlinematerial 1: Leitfaden für Einzelinterviews und Fokusgruppen mit bayerischen Hausärzt*innen bezüglich des Verordnungsverhaltens vor dem Hintergrund der Bayerischen Wirkstoffvereinbarung im Befragungszeitraum 11/2019 – 03/2020. 2

Onlinematerial 2: Tabelle mit Kategoriensystem zur Auswertung der Interviews und Fokusgruppen mit bayerischen Hausärzt*innen bezüglich des Verordnungsverhaltens vor dem Hintergrund der Bayerischen Wirkstoffvereinbarung im Befragungszeitraum 11/2019 – 03/2020. 7

Onlinematerial 1: Leitfaden für Einzelinterviews und Fokusgruppen mit bayerischen Hausärzt*innen bezüglich des Verordnungsverhaltens vor dem Hintergrund der Bayerischen Wirkstoffvereinbarung im Befragungszeitraum 11/2019 – 03/2020.

Anmerkungen:

Pro Interview werden ca. zwei Vignetten vorbereitet und je nach Fachgebiet konstruiert.

* Gelb markierte Fragen wurden ausschließlich in den Einzelinterviews gestellt.

1. Allgemeine Informationen zu Person und Praxiserfahrung

Zunächst möchten wir Sie bitten, sich der Reihe nach vorzustellen. Das hilft uns natürlich, um Sie alle kennenzulernen und zugleich ist es für die Verschriftlichung später wichtig, eine Sprachaufnahme von jedem von Ihnen zu Beginn zu bekommen.
**Stellen Sie sich mit ein paar Worten vor und erzählen Sie uns doch, wo Ihre Praxis ist und wie typische Patientenfälle bei Ihnen aussehen.**
- Zum Beispiel Alter, Geschlecht, Krankheitsbild, Verhalten

2. Allgemeines zum Verordnungsverhalten

Nun geht es um Therapieoptionen und Medikationsverordnungen.

Bsp.: Ganz allgemein, wenn ein Patient in Ihre Praxis kommt, Sie eine Diagnose stellen und er ein Medikament bekommen soll:

Welche Kriterien sind für Sie wichtig, wenn Sie zwischen verschiedenen Arzneimitteltherapien auswählen?

Beispiele vorlegen, nachdem der Arzt seine ersten Antworten erklärt hat:

- Wirkmechanismus
- Wirkung
- Vorgabe der Leitlinien
- Hersteller
- Preise
- Persönliche Erfahrungswerte
- Pharmavertreter

- Fortbildungen
- Kongresse
- Patientenwünsche
- ehemalige Regressansprüche
- Begleitmedikationen
- Komorbidität
- Studien

Inwiefern haben Patienten und ihre Wünsche Einfluss auf Ihre Arzneimittelverordnung?

- *Was für Wünsche sind das zum Beispiel?*
- *Wie gehen Sie damit im Alltag um? (Stichwort: Aut idem)*
- *Wie reagieren Ihre Patienten, wenn Sie nicht das Präparat verschreiben, welches der Patient haben möchte? (Stichwort: Adhärenz-Probleme)*
- *Wie oft kommt es vor?*

Wenn sich bei einem Medikament konkrete Fragen ergeben, wie gehen Sie dann vor?*

Hier vor allem: Wirksamkeit, Sicherheit, Wirtschaftlichkeit – wie informieren Sie sich?

Es werden ja regelmäßig neue Medikamente auf dem Markt zugelassen, bei denen Sie entscheiden müssen, ob Sie sie Ihren Patienten geben oder nicht. **Wie gehen Sie dabei vor?**

Und wie gehen Sie bei vom Krankenhaus/von anderen Fachärzten initiierten Therapien vor?

- *Welche Auswirkungen haben von anderen Fachärzten initiierte Therapien auf Ihr Verordnungsverhalten?*
- *Werden diese weitergeführt?*

3. Gesamtsituation und Bayerische Wirkstoffvereinbarung

Es gab einen Artikel von der Kassenärztliche Vereinigung (KV) Hamburg, der diese Art von Wirkstoffvereinbarung zusammenfassend wie folgt beschreibt:

Im ehemaligen System der Richtgrößenprüfung hatten die Ärzte die Verantwortung für die Arzneimittelkosten – ein grundlegender Konstruktionsfehler.

Bei dem neuen Modell geht es nicht mehr um absolute Kosten, sondern um definierte Tagesdosen (DDD)-Quoten und Anteile von Generika und Leitsubstanzen.

Mit Wendung: Der Arzt verantwortet lediglich, was er selbst beeinflussen kann - er wählt den richtigen Wirkstoff aus.

„Die eigentliche Kostenfrage verschiebt das Modell weg von den Ärzten und dahin, wo sie hingehört, nämlich zu den Krankenkassen“

Inwiefern passt das zu den Erfahrungen, die Sie mit der Bayerischen Wirkstoffvereinbarung gemacht haben? Wie beurteilen Sie diese Aussage?

- *Inwiefern hat sich an Ihrem Verordnungsverhalten seitdem etwas verändert?*
- *Welche Auswirkungen nehmen Sie von der Bayerischen Wirkstoffvereinbarung auf Ihre ärztliche Therapiefreiheit/Therapiehoheit wahr?*
- *Vorteile/Nachteile zu früheren Verfahren?*
- *Mit welchen Adjektiven kann man sie beschreiben? (Positive wie negative Beschreibungen; einschränkend, hilfreich, unterstützend, absichernd)*
- *Gibt es Erkrankungen/Indikationen, die Sie anders therapieren würden, als es die Wirkstoffvereinbarung vorgibt?*
- *Wie schaffen Sie es, Ihre Ziele im Rahmen der Bayerischen Wirkstoffvereinbarung einzuhalten?*
    - *Haben Sie schon einmal ein Beratungsangebot zur Verordnungshilfe wahrgenommen?*
    - *Wenn ja, wie lief dieses ab?*

Wie zufrieden sind Sie aktuell/jetzt im Moment mit Ihrer Verordnungssituation?

- *Wie schätzen Sie die Zufriedenheit Ihrer Kollegen/anderer Fachärzte mit der Verordnungssituation ein?*

Was erwarten Sie von der Kassenärztlichen Vereinigung Bayerns (KVB)? Wird die KV diesen Erwartungen gerecht?

Was würde Ihnen das Verordnen erleichtern? Wie könnte man das System der bayerischen Wirkstoffvereinbarung verbessern?

Die Bayerische Wirkstoffvereinbarung wurde ja aus einem bestimmten Grund eingeführt und basiert auf den Paragrafen zum Wirtschaftlichkeitsgebot (Paragrafen vorlegen). **Wo führt Ihrer Meinung nach diese Entwicklung in den nächsten fünf bis zehn Jahren hin?**

Vorlegen der Paragrafen:

§ 12 Fünftes Buch Sozialgesetzbuch (SGB V) Wirtschaftlichkeitsgebot

Die Leistungen müssen ausreichend, zweckmäßig und wirtschaftlich sein; sie dürfen das Maß des Notwendigen nicht überschreiten. Leistungen, die nicht notwendig oder unwirtschaftlich sind, können Versicherte nicht beanspruchen, dürfen die Leistungserbringer nicht bewirken und die Krankenkassen nicht bewilligen.

§ 106 SGB V Wirtschaftlichkeitsprüfung

Die Krankenkassen und die Kassenärztlichen Vereinigungen überwachen die Wirtschaftlichkeit der vertragsärztlichen Versorgung durch Beratungen und Prüfungen.

In der medizinischen Versorgung wird viel von Kosten und Wirtschaftlichkeit gesprochen, die auch laut Gesetz überprüft werden soll. Wie geht es Ihnen eigentlich persönlich damit?*

- *Arbeitsmotivation*
- *Emotion*

Sind in Ihren Augen im Bereich der Arzneimitteltherapie die Begriffe „Wirtschaftlichkeit“ und „Qualität“ vereinbar?

4. Abschluss & Fazit

Zuletzt möchten wir noch Raum für weitere Aspekte geben, die Ihnen möglicherweise noch wichtig sind.

Was war Ihre Motivation hier bei uns teilzunehmen?*

Gibt es denn weitere Aspekte, welche Sie gerne noch ansprechen möchten?

Nachdem wir diese verschiedenen Facetten beleuchtet haben, möchten wir gerne ein Fazit ziehen.

Wenn Sie jetzt an Ihre Praxisarbeit denken, welche Aspekte sehen Sie kritisch/Was würden Sie gerne ändern, wenn Sie könnten?*

Und was bereitet Ihnen besonders Freude?*

Ausklang: Herzlichen Dank für die Teilnahme!

<u>Onlinematerial 2: Tabelle mit Kategoriensystem zur Auswertung der Interviews und Fokusgruppen mit bayerischen Hausärzt*innen bezüglich des Verordnungsverhaltens vor dem Hintergrund der Bayerischen Wirkstoffvereinbarung im Befragungszeitraum 11/2019 – 03/2020.</u>

Anmerkung: Das Kategoriensystem ist ein Teilausschnitt mit den für den Artikel relevanten Kategorien.

Hauptkategorie	Generische Kategorie	Unterkategorie	Beschreibung
**Wirtschaftlichkeit**	Arztverständnis von Wirtschaftlichkeit	Beurteilung des Wirtschaftlichkeits-gebots	Umfasst Aussagen, die Aufschluss darüber geben, welches Arztverständnis von Wirtschaftlichkeit vorliegt. Wird insbesondere die eigene Wirtschaftlichkeit der Praxis erläutert oder die Gesamtwirtschaftlichkeit bezogen auf das Gesundheitssystem?
	Therapien werden weitergeführt: Hausärzt*innen mit geringen Regresssorgen	Dokumentation	Umfasst Aussagen der Hausärzt*innen, welche die Weiterführung von Therapien damit begründen, dass man sich im Falle eines Prüfverfahrens bezüglich der Wirtschaftlichkeit durch eine sorgfältige, schriftliche Dokumentation der Verordnung absichern könne.
	Therapien werden weitergeführt: Resignierte	Resignation	Umfasst Aussagen der Hausärzt*innen, welche der Meinung sind, dass die widersprüchlichen Anforderungen nicht vereinbar sind und Aussagen zu dem daraus resultierenden Verhalten. Dies beinhaltet Aussagen zu chronischer Hilflosigkeit und Vernachlässigung von Wirtschaftlichkeit.
	Therapien werden nicht weitergeführt: Hausärzt*innen mit größeren Regresssorgen	Gleichwertige Alternativen	Beinhaltet Aussagen, welche die Entscheidung zur Umstellung von Therapien damit begründen, dass es zu einigen teuren Medikamenten, welche von Krankenhausärzt*innen verschrieben werden, auch gleichwertige, günstigere Alternativen gibt.

Hauptkategorie	Generische Kategorie	Unterkategorie	Beschreibung
		Priorisierung von Wirtschaftlichkeit	Beinhaltet Aussagen, die eine Umstellung der Therapie, die von Krankenhausärzt*innen verordnet wurden, damit begründen, dass Hausärzt*innen bestimmte Richtlinien bezüglich der Medikamentenvergabe einhalten müssen und deshalb die verordneten Therapien nicht weiterführen.
**Wirtschaftlich herausfordernde Medikamenten-gruppen**	Auswirkungen auf die Trendmeldungen der Wirkstoff-vereinbarung	Medikamente zur Behandlung von Rheuma<br><br>Medikamente zur Behandlung von Herzinsuffizienz<br><br>Medikamente zur Behandlung von arterieller Hypertonie<br><br>Medikamente zur Behandlung von COPD/Asthma<br><br>Psychopharmaka<br><br>Diabetes-Therapeutika<br><br>Orale Antikoagulantien	Beinhaltet Aussagen zum Umgang mit wirtschaftlich herausfordernden Medikamentengruppen zur Behandlung bestimmter Krankheitsbilder wie Rheuma, Herzinsuffizienz, arterielle Hypertonie, COPD/Asthma, Psychopharmaka, Diabetestherapie, orale Antikoagulantien und die Auswirkungen auf die Trendmeldungen der Wirkstoffvereinbarung (grüne, gelbe oder rote Ampel).
**Schnittstellen-problematik**	Übergang stationär/ ambulant	Unterschiedliche Anforderungen zwischen Sektoren	Umfasst Aussagen, die Aufschluss darüber geben, welche Schwierigkeiten bestehen und entsprechend verbessert werden müssten.
	Zusammenarbeit mit Kolleg*innen	Kommunikation	Umfasst Aussagen, die Aufschluss darüber geben, ob und in welcher Form ein Austausch zwischen Hausärzt*innen und Krankenhausärzt*innen stattfindet.

Hauptkategorie	Generische Kategorie	Unterkategorie	Beschreibung
**Umgang mit Therapien von Kolleg*innen**	Therapien werden weitergeführt: Ärzt*innen-zentrierte Faktoren	Vertrauen	Umfasst Aussagen der Hausärzt*innen, die wegen des Vertrauens in die Kompetenz der Krankenhausärzt*innen eine Weiterführung der Therapien begründen.
		Indikation	Beinhaltet Aussagen, die durch Indikation die Weiterführung der Therapie als angemessen betrachten und deshalb die Medikamente, die von Krankenhausärzt*innen verordnet wurden, weiterverschreiben.
		Keine Honorierung für Aufwand	Umfasst Gründe für die Weiterführung der Therapien, die den Zeitaufwand betreffen. Da eine Umstellung der Medikamente und der damit verbundene (Zeit-)Aufwand nicht honoriert werden, werden diese weiterverordnet.
	Therapien werden weitergeführt: Patient*innen-zentrierte Faktoren	Widerstand	Umfasst Aussagen, welche eine Weiterführung der Therapie durch den Widerstand von Patient*innen begründen, wenn Hausärzt*innen empfehlen, die Medikamente, die von den Krankenhausärzt*innen verordnet wurden, auf gleichwertige Alternativen umzustellen. Da Patient*innen teure Medikamente oft mit „guten“ Medikamenten gleichsetzen, leisten diese bei dem Vorschlag einer Umstellung durch den Hausarzt Widerstand.
		Praxislage (*nicht im Artikel)	Beinhaltet Aussagen, welche als Grund für das Weiterführen der Therapien den Praxisstandort anführen. Da der Weg zu Facharztpraxen häufig weit ist, verordnen Hausärzt*innen Medikamente weiter.

Hauptkategorie	Generische Kategorie	Unterkategorie	Beschreibung
	Therapien werden nicht weitergeführt: Ärzt*innen-zentrierte Faktoren	Vertrauen in Kolleg*innen	Beinhaltet Meinungsäußerungen, die ein fehlendes Vertrauen der Hausärzt*innen gegenüber der Kompetenz der Krankenhausärzt*innen zeigen, aufgrund dessen die von diesen verordneten Therapien nicht weitergeführt werden.
		Indikation	Umfasst Aussagen, die die fehlende Indikation für die von den Krankenhausärzt*innen verordneten Medikamente als Grund dafür anführen, die Therapie nicht weiterzuführen.
		Studienlage (*nicht im Artikel)	Umfasst Aussagen, welche die Studienlage als Grund dafür anbringen, die von den Krankenhausärzt*innen verordneten Therapien nicht weiterzuführen.
		Eigene Kompetenz	Umfasst Aussagen, die die Umstellung von Medikamenten mit der eigenen Kompetenz begründen. Durch das Hinterfragen der verschriebenen Therapie der Krankenhausärzt*innen durch die Hausärzt*innen kommt es zur Entscheidung, die Medikamente nicht weiterzuverordnen.
		Krankenhaus ist andere Situation	Beinhaltet Aussagen, welche die Therapieumstellung damit begründen, dass das Krankenhaus eine andere Situation darstellt als eine Hausarztpraxis. Therapien, welche in der Klinik sinnvoll sind, können nach der Entlassung nicht mehr angemessen oder zweckmäßig sein und werden durch die Hausärzt*innen entsprechend angepasst.
